# Case Report: Giant Colonic Lipoma, a Rare Benign Tumor Mimicking Malignancy

**DOI:** 10.3390/reports8010021

**Published:** 2025-02-11

**Authors:** Bogdan Oprita, Ioana Adriana Serban, Valentin Enache, Alina Prodan, Ruxandra Oprita

**Affiliations:** 1 Faculty of Medicine, University of Medicine and Pharmacy “Carol Davila”, 050474 Bucharest, Romania; bogdan.oprita@umfcd.ro (B.O.); ruxandra.oprita@umfcd.ro (R.O.); 2 Emergency Department, Clinical Emergency Hospital of Bucharest, 105402 Bucharest, Romania; 3 Romania Gastroenterology Department, Clinical Emergency Hospital of Bucharest, 105402 Bucharest, Romania; 4 Pathology Department, Clinical Emergency Hospital of Bucharest, 105402 Bucharest, Romania; valienache00@gmail.com; 5 Surgery Department, Clinical Emergency Hospital of Bucharest, 105402 Bucharest, Romania; alina.prodan@yahoo.co.uk

**Keywords:** lipoma, giant colonic lipoma, lipoma of the hepatic flexure, computed tomography, endoscopy

## Abstract

**Background and Clinical Significance:** Colonic lipomas are benign tumors composed of adipose tissue, frequently asymptomatic and found incidentally on routine surveillance. Most lesions are smaller than 2 cm in diameter, while giant lipomas are characterized as over 4 cm. Giant colonic lipomas, though rare, may present with obstructive symptoms, gastrointestinal bleeding or intussusception and can mimic malignant lesions on imaging and endoscopic examination. Historically, the resection of these lesions has been limited to those that are larger or symptomatic. Recent observations indicate that lipomas may retain growth potential and can become symptomatic over time, though being inconsequential initially. Surgical resection is favored over endoscopic excision for lipomas above 2 cm to mitigate risks including haemorrhages and perforations. **Case Presentation**: We report a case of a 38-year-old female who exhibited non-specific gastrointestinal symptoms with a high suspicion of malignancy based on imaging and endoscopy but was ultimately diagnosed with a benign giant colonic lipoma. **Conclusions:** This case presents the challenges in diagnosing giant colonic lipomas, which, in certain cases, can mimic malignant lesions. Histopathological analysis remains the gold standard for confirming the diagnosis, especially in cases with atypical features.

## 1. Introduction and Clinical Significance

Lipomas are benign mesenchymal tumors located in the subcutaneous tissue, consisting of adipose tissue encased in a fibrous layer [1]. Within the gastrointestinal system, they are predominantly located in the small intestine, stomach, and esophagus [2]. Colonic lipomas are rare benign tumors; incidence may be as high as 4.4% but is more likely to be less than 1% in the colon and rectum [3]. They are usually asymptomatic and discovered incidentally, with most lesions being small (under 2 cm) and frequently located in the submucosal layer of the right colon. Giant colonic lipomas, defined as those larger than 4 cm, are associated with a range of clinical symptoms due to their size and potential to obstruct the colonic lumen or cause mucosal ulceration. Epidemiologically, giant colonic lipomas predominantly affect middle-aged to elderly adults, with a higher prevalence in females [3]; they are typically solitary but, rarely, they can present as multiple or diffuse lesions [4]. Giant lipomas result from abnormal proliferation of submucosal adipose tissue and can extend into the muscularis propria. The considerable dimensions of these lesions often exert mechanical pressure on the overlaying mucosa resulting in mucosal ischaemia and ulceration which can induce local inflammation, further complicating the case by generating areas of reactive tissue change and desmoplastic reaction, potentially resembling malignancy. This case contributes to the literature by outlining the diagnostic and therapeutic challenges posed by giant colonic lipomas, especially their ability to clinically and radiologically resemble malignancy. Each reported case, due to its rarity, contributes to our understanding of differential diagnoses and informs management strategies. This case report aims to offer insights into the diagnostic process, clinical decision-making, and effective management strategies.

## 2. Case Presentation

A 38-year-old female presented to the emergency department with complaints of intermittent abdominal pain, abdominal distension, and alterations in bowel habits, specifically alternating constipation and diarrhea. Her past medical history was unremarkable, with no previous gastrointestinal conditions or surgeries. She was subsequently admitted to the gastroenterology department for further investigation.

Initial laboratory results, including complete blood count, coagulation profile, liver and kidney function tests, were all within normal limits.

**Figure 1:**
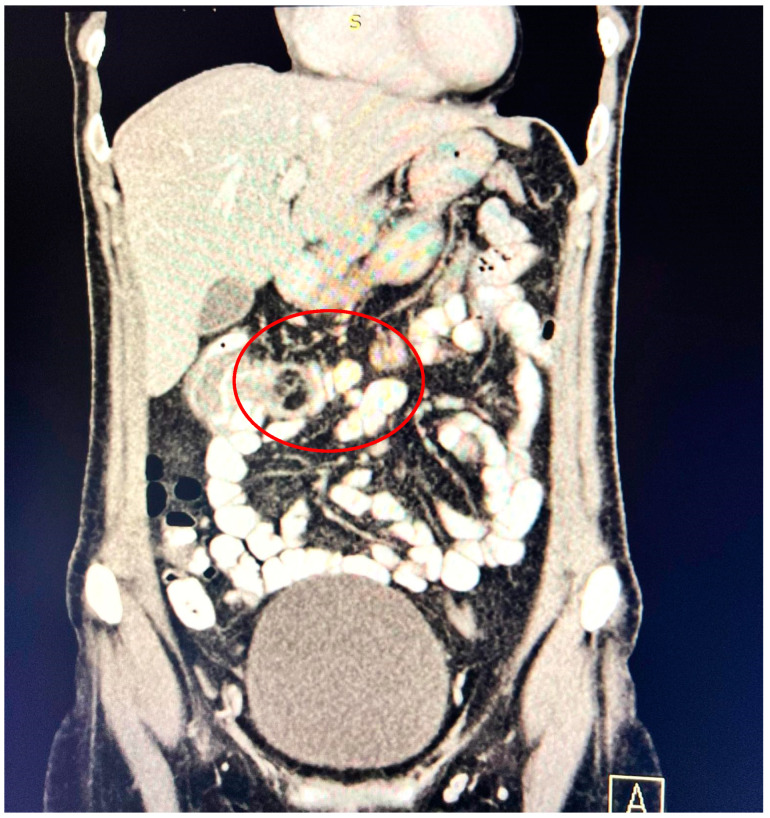
Circumferential parietal thickening noted at the hepatic flexure of the colon measuring 21/45 mm, moderately iodophilic, associated with discrete linear-type densification of locoregional fat, indicative of a desmoplastic reaction.

**Figure 2:**
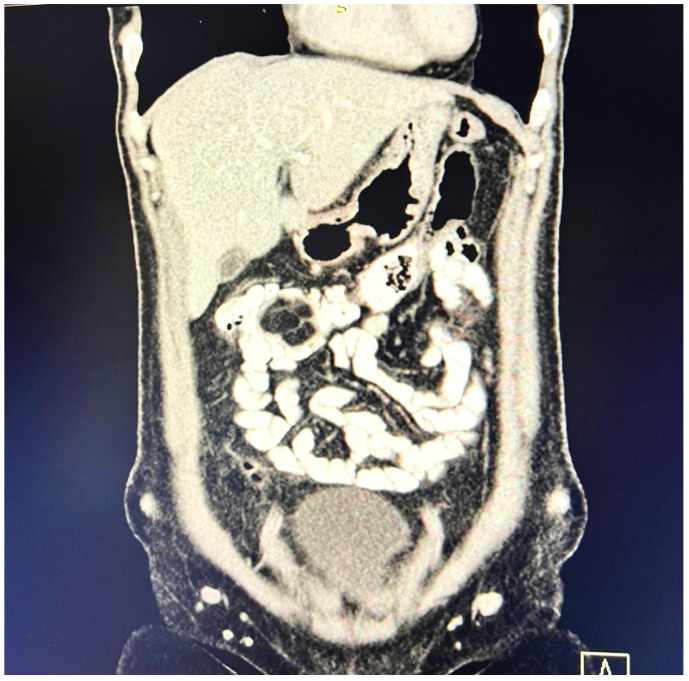
Intralumenal mass with an axial diameter of 29/28 mm and extended cranio-caudally over a length of 27 mm, showing polycyclic contour, discretely iodophilic peripherally.

**Figure 3:**
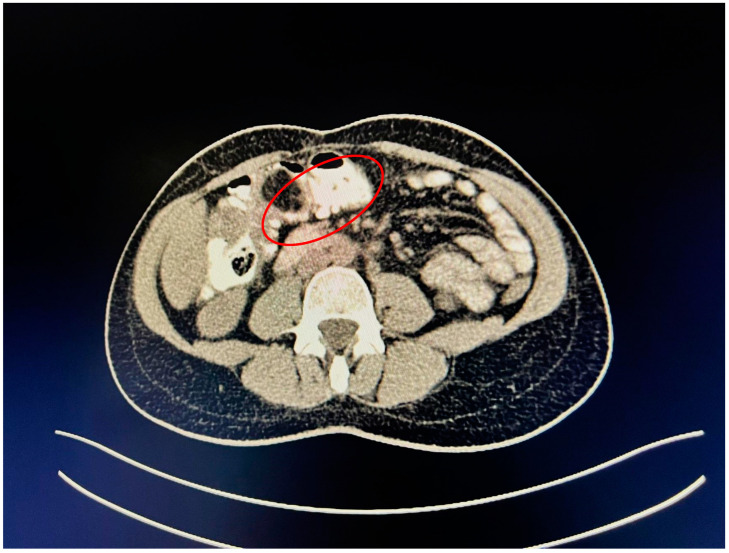
Mass resulting in filiform axial narrowing of the lumen without associated distention of the colon.

A contrast-enhanced computed tomography (CT) scan conducted on the day of admission revealed circumferential parietal thickening of a maximum of 21 mm at the hepatic flexure of the colon. This thickening extended over a length of approximately 45 mm, resulting in filiform, axial stenosis of the lumen, without any overlying distension of the colonic frame. The parietal thickening was moderately iodophilic and accompanied by discrete linear-type densification of locoregional fat, which was most likely a desmoplastic reaction. A tumor formation with a maximum axial diameter of approximately 29/28 mm, extending craniocaudal on a length of approximately 27 mm, was located at the distal end of the parietal ingrowth with an intraluminal site. This tumor formation had a polycyclic contour, thin septa within, and a discrete lodophilic peripheral appearance. It appeared to have a wide base of implantation at the level of the postero-inferior wall of the colon; mild hepatomegaly in the liver, with a homogeneous structure and a regular surface, and the absence of focal primary or secondary lesions; adenopathies in the hepatic hilum with a maximum diameter of 16/12.5 mm and in the celio-mezenteric territory with a maximum diameter of 24/19 mm; microadenopathies with the gastro-hepatic ligament; and an absence of subdiaphragmatic fluid (Figure 1, Figure 2 and Figure 3).

**Figure 4:**
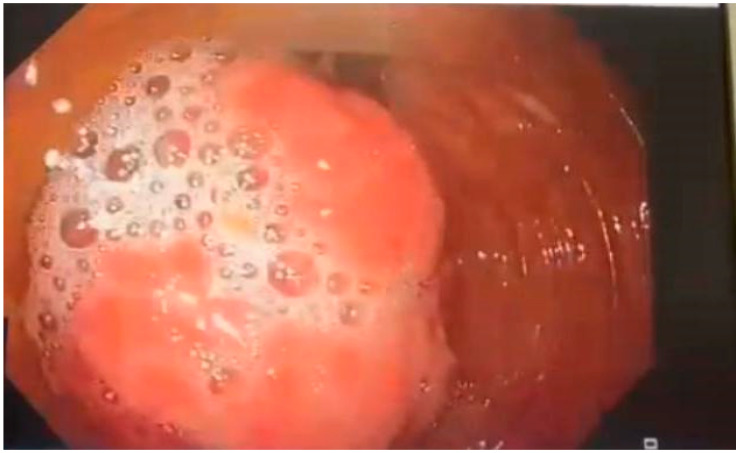
Polypoid mass at the hepatic flexure occupying more than 75% of the luminal circumference.

**Figure 5:**
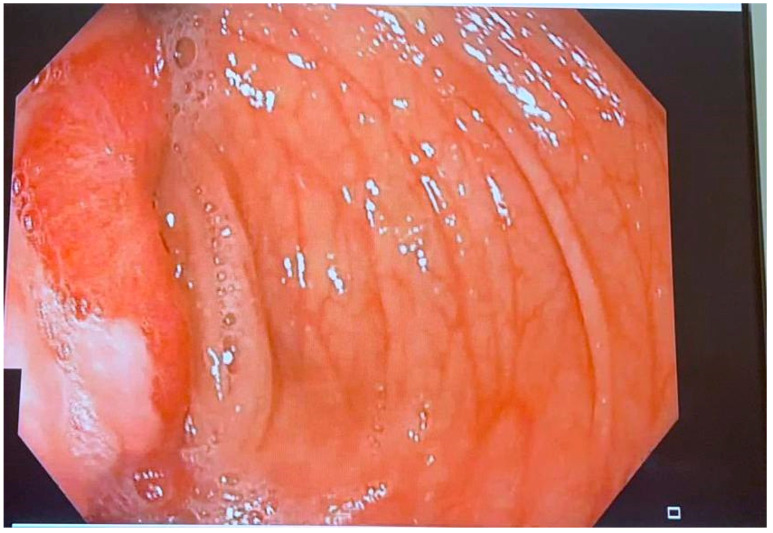
Polypoid mass with central ulceration.

A colonoscopy performed the following day showed a large hyperemic round tumor-like mass approximately 3.5 cm in diameter, located at the hepatic flexure. The lesion displayed a central ulceration and erythematous mucosa, occupying more than three-quarters of the luminal circumference suggesting malignant gastrointestinal stromal tumor (GIST). The tumor was firm on examination, had a negative pillow sign, and allowed only slight advancement of the colonoscope due to its size. Bite-on-bite biopsies were taken, presenting a negative “naked fat sign” and the lesion was tattooed for localization during subsequent surgical intervention (Figure 4 and Figure 5). The biopsy results obtained during the colonoscopy came back after 96 h and were negative for malignancy.

**Figure 6:**
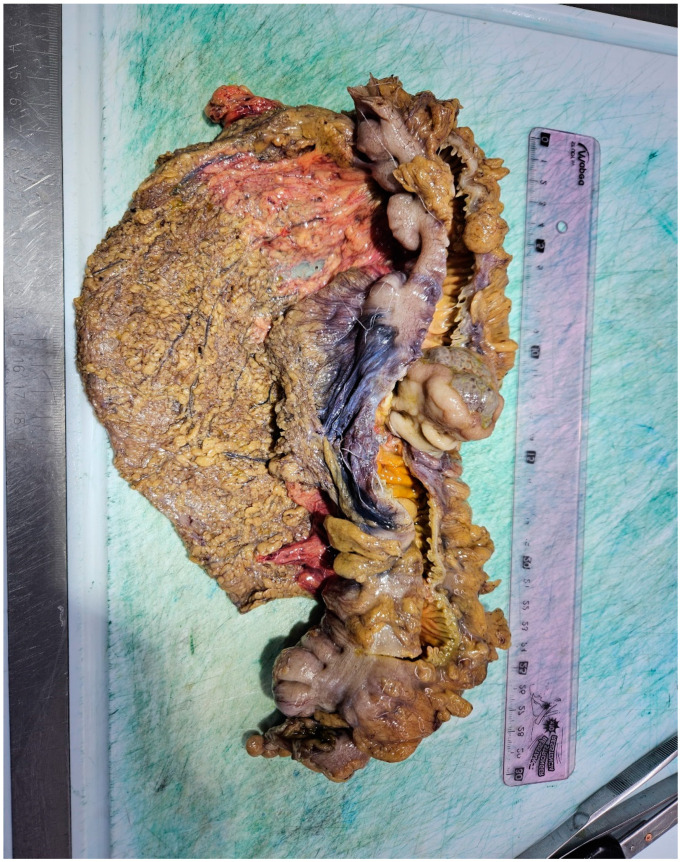
Right hemicolectomy revealing polypoid tumor partially ulcerated.

**Figure 7:**
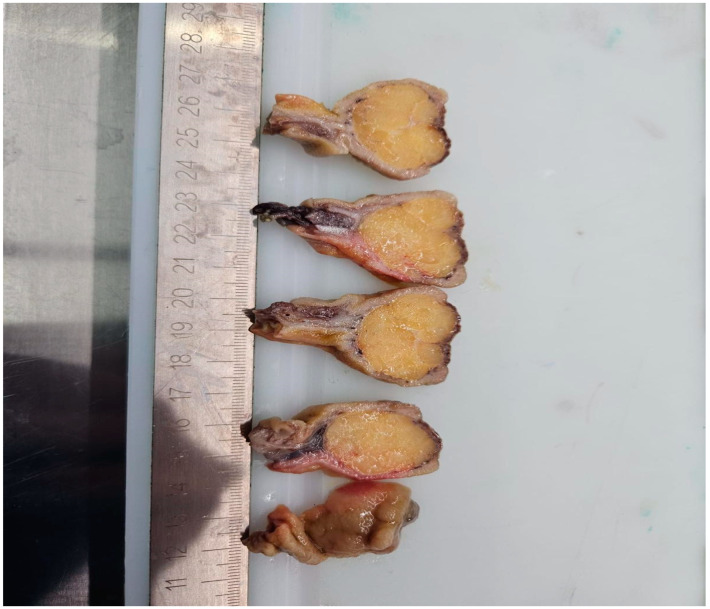
Macroscopic examination of the polypoid tumor measuring 4 × 3 × 3 cm.

Given the suspicion of malignancy and the clinical presentation, the patient was referred to the surgery department and underwent a right hemicolectomy the following day. Macroscopic examination of the resected specimen revealed a partially ulcerated polypoid tumor measuring 4 × 3 × 3 cm, with a broad implantation base of 2.5 × 2 cm (Figure 6 and Figure 7).

**Figure 8:**
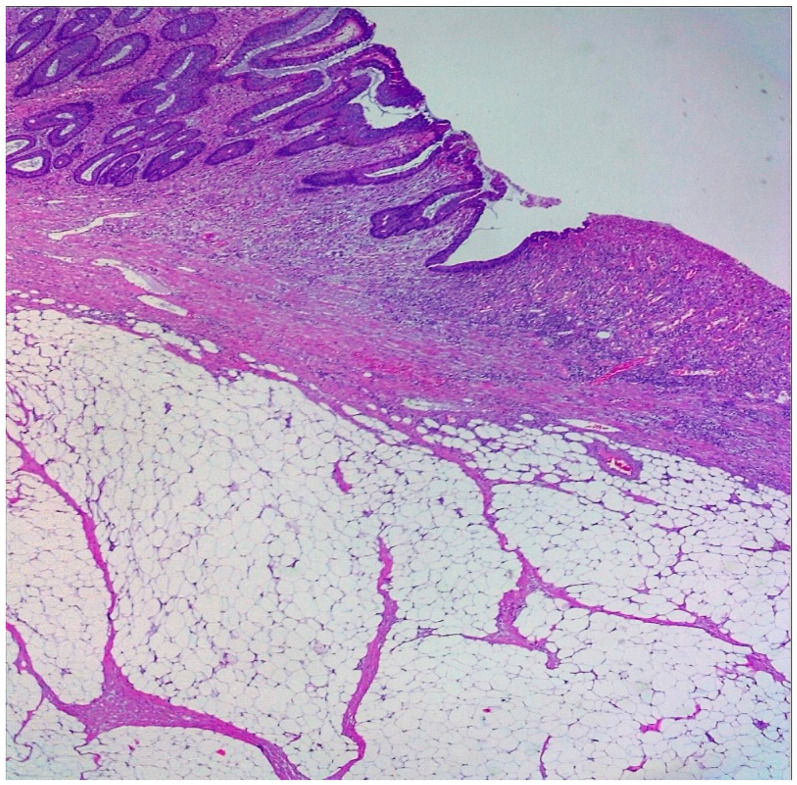
H&E stain 50× Submucosal lipoma with ulcerated colonic mucosa.

Histopathologic examination confirmed a benign polypoid submucosal lipoma with areas of ulceration and no significant cellular atypia, mitotic figures, or pleomorphism evidence of malignancy (Figure 8). The patient had an uncomplicated postoperative course and was discharged in stable condition one week later.

At the three-month follow-up, the patient remained clinically stable and asymptomatic, with no recurrence of gastrointestinal symptoms.

The decision to proceed with a right hemicolectomy was based on the high clinical and radiological suspicion of malignancy. Given the tumor’s size, its ulcerated appearance, and the significant luminal narrowing raising concerns for potential obstruction and the presence of desmoplastic reaction on imaging, malignancy could not definitively be ruled out preoperatively. Alternative approaches, such as endoscopic resection, were not considered viable due to the lesion’s size, broad implantation base, and the absence of a clear margin separating it from deeper colonic layers. Laparoscopic-assisted techniques were theoretically an option, but, given the high suspicion of malignancy and the need for oncologic resection, an open right hemicolectomy was deemed the most appropriate course of action.

This case presented a significant diagnostic challenge due to the contrast between the lesion’s suspicious appearance on imaging and endoscopy and its ultimately benign nature confirmed by histopathology. The presence of central ulceration and erythema further complicated the assessment, as these features are more commonly linked to malignancies than benign lipomas. Preoperative imaging played a key role in guiding clinical decisions. While advanced techniques such as MRI with fat suppression or endoscopic ultrasound could have helped distinguish adipose tissue from neoplastic changes, the lesion’s location at the hepatic flexure and its size made endoscopic ultrasound impractical. Although imaging provided valuable information, it also contributed to diagnostic uncertainty, emphasizing the importance of histopathological confirmation in such cases.

## 3. Discussion

Colonic lipomas originate from mesenchymal tissue and are primarily located on the right side of the colon, either in the submucosal or subserosal layers [5]. Prior findings have indicated a size variety from 0.35 to 10 cm in diameter, with merely 30% exceeding 2 cm [6]. Colonic lipomas tend to be solitary but in 10–20% can be multiple. The age at diagnosis ranges within 50–70 years, and is more commonly seen in women [7]. Most lipomas are asymptomatic and are typically discovered inadvertently during surveillance colonoscopy, CT imaging, or autopsy. Symptoms correlate with the size of the lipoma, often presenting with vague symptoms, including abdominal pain, alterations in bowel habits (such as alternating constipation and diarrhea), abdominal distension, and, in some cases, hematochezia or occult gastrointestinal bleeding [7]. Giant lipomas, particularly those in the right colon, may even present with signs of partial or complete bowel obstruction and are the most common benign tumors in the colon that cause intussusception [3]. Although colonic lipomas are relatively uncommon and often asymptomatic, accurate identification and diagnosis are essential for appropriate management, as they may mimic gastrointestinal stromal tumors [8], contain premalignant lesions (tubulovillous adenomas), or aid in the detection of genetic syndromes (Cowden) [9]. Despite lacking malignant potential, lipomas can occasionally be challenging to differentiate from liposarcomas or other malignant lesions. Liposarcoma is a locally aggressive mesenchymal tumor composed of mature adipocytes and stromal cells with at least focal cytologic atypia with the following essential features: low-grade lipogenic tumor with multiple morphologic subtypes and significant histologic variability, molecularly characterized by ring or giant marker/rod chromosomes composed of material from 12q13-15, and the differential diagnosis is accomplished by the amplification of the murine double minute-2 (MDM2) gene [10]. Lipomas are benign entities with no risk of malignant transformation, however atypical lipomatous tumors (ALTs), also known as well-differentiated liposarcomas (WDLs), are considered premalignant and have a risk of local recurrence between 10–50% over 10 years [11]. The diagnosis of a sarcoma requires an inquiry into its potential association with an inherited genetic disorder, including nevoid basal cell carcinoma syndrome (Gorlin syndrome), familial adenomatous polyposis (Gardner syndrome), Li-Fraumeni syndrome, tuberous sclerosis (Bourneville disease), and neurofibromatosis type 1 (von Recklinghausen’s disease) [12].

Diagnosing colonic lipomas, especially giant ones, requires a combination of imaging and endoscopic evaluation. Lipomas typically appear as well-demarcated, homogenous, hypodense masses on computed tomography (CT), often with fat density characteristics. Although, larger lipomas may exhibit surrounding desmoplastic reaction and regional adenopathy. Endoscopy can provide visual and tactile clues, with features such as the “pillow sign” (indentation on pressure) and “naked fat sign” (protrusion of yellow fat upon biopsy) indicating lipomatous nature [13]. Nevertheless, endoscopic appearance alone is insufficient to rule out malignancy due to the potential for mucosal ulceration and obstructive features. Histopathologic examination, therefore, remains the gold standard for diagnosing colonic lipomas. Microscopic findings include mature adipocytes with clear cytoplasm and eccentric, compressed nuclei, a thin fibrous capsule may surround the lipoma, no significant cellular atypia, mitotic figures or pleomorphism, mild chronic inflammation if ulceration or mucosal erosion has occurred, and, in some cases, smooth muscle fibers from the muscularis propria may be present, A thorough pathological assessment following resection is essential, particularly for atypical lipomas with irregular morphology or accelerated growth. Previously, endoscopic treatment of lipomas was restricted to symptomatic lesions exhibiting favorable morphological traits (pedunculated) and measuring less than 2 cm in diameter [14] As endoscopic devices and techniques have advanced, endoscopists have increasingly been able to resect colonic lipomas exhibiting a broader range of features.

Bronswijk et al. [15] conducted a systemic review evaluating four distinct endoscopic techniques for managing colonic lipomas: lipoma unroofing, dissection-based resection-endoscopic submucosal dissection (ESD) and stalk dissection, endoscopic mucosal resection (EMR) either en bloc or piecemeal, with or without submucosal injection, and loop-assisted snare techniques. While each method demonstrated comparable clinical remission rates, notable differences were observed in terms of endoscopic resolution and adverse event profiles. Lipoma unroofing, which involves partial snare resection of the overlaying mucosa to facilitate the spontaneous intraluminal expulsion of the adipose tissue, exhibited the lowest rate of adverse events. However, its efficacy in achieving complete lesion resolution was limited (60%). Studies suggest that incomplete resection may require additional procedures, particularly for larger lesions. Despite these concerns, clinical symptom relief was comparable to that achieved through more invasive techniques. Dissection-based techniques, including ESD and stalk dissection, were associated with higher rates of complete lesion resolution due to their ability to achieve en bloc resection (93.6%). However, these procedures are technically demanding and require specialized training, limiting their widespread applicability in routine clinical practice. Similarly, EMR and loop-assisted resections demonstrated higher rates of endoscopic resolution (93.1%) but were associated with a higher prevalence of adverse events such as post-procedural bleeding and perforation.

The review identified significant methodological variability across the studies examined, which included case reports, retrospective analyses, and one prospective trial. The heterogeneity hindered the feasibility of conducting a formal meta-analysis and limited the generalizability of the findings. The lack of long-term follow-up data raises concerns regarding the sustainability of symptom relief and the possibility of lesion recurrence after unroofing. Given the scarcity of large-scale comparative studies and the lack of randomized controlled trials, clinical decision-making should remain individualized. It has been proposed that, pending the availability of larger studies or extended follow-up data, the care of symptomatic large and giant colon lipomas should be tailored according to local expertise, the patient’s age, and co-morbid diseases [16].

Jiang and colleagues [17] assert that surgical intervention is indicated when the lipoma exceeds 4 cm in size; when the preoperative diagnosis is uncertain; when the lipoma is associated with intussusception and the patient exhibits symptoms; when there is involvement of the muscular or serosal layer; or when the lesion is not suitable to endoscopic resection. The selection of surgical intervention approaches is contingent upon the size and location of the lipoma, as well as the existence of comorbidities. Surgical treatment is required for lipomas exceeding 4 cm in size that cannot be excised endoscopically or in cases of complications such as intestinal obstruction due to intussusception (the most common complication, particularly in the small intestine, though with a low incidence in adults—2–3 cases per 1,000,000 population/year) [18] and intraluminal stenosis caused by the lipoma, mesenteric ischemia, or intestinal necrosis. Various surgical techniques can be employed depending on the location of the lesion, its size, associated complications, the patient’s age and comorbidities, and their clinical–biological status. In cases where intestinal obstruction or an acute complication is excluded, laparoscopic or robot-assisted approaches are preferred [19]. Conversely, in emergency cases or in the absence of minimally invasive services, laparotomy is the preferred approach. If the lesion is relatively accessible and the diagnosis of a giant lipoma is nearly certain—thus excluding the possibility of malignancy—enucleation may be performed either laparoscopically [20] or through open laparotomy. The lesion is identified as a homogenous, well-demarcated, soft, depressible mass located under the serosa or within the colonic wall. A longitudinal incision is made, followed by dissection to separate the serosa from the submucosa. The mass is dissected with minimal impact on the mucosa and muscular layers, and the stalk, if present, is ligated. Hemostasis is achieved through electrocauterization. The procedure is completed with enterorrhaphy using absorbable sutures, ensuring sufficient tension to avoid wound dehiscence but not so much as to create excessive strain on the sutures. A “saline leak” test is performed, followed by the closure of the surgical defect. Alternative surgical techniques are segmental colectomy, hemicolectomy, or total colectomy may be necessary, particularly in cases of intestinal obstruction complicated by significant ischemia and extensive areas of necrosis. Segmental colectomy, as performed in this case, is often recommended when malignancy cannot be excluded based on imaging and biopsy findings. The recurrence of colon lipoma following surgical intervention has not been recorded.

## 4. Conclusions

Colonic lipomas are benign tumors consisting of adipose tissue, often asymptomatic and discovered incidentally during routine surveillance. The majority of lesions are small and asymptomatic; nevertheless, giant colonic lipomas are linked to various clinical manifestations due to their size and potential to obstruct the colonic lumen or cause mucosal ulceration. The treatment of choice for symptomatic lipomas is resection of the tumor, either surgical or endoscopic, which yields a favorable prognosis if the tumor is entirely excised. Future studies could focus on improving preoperative diagnosis, especially by utilizing advanced imaging methods, such as MRI with fat suppression, which may help distinguish benign lipomas from malignancies more accurately.

## Data Availability

The original data presented in the study are included in the article, further inquiries can be directed to the corresponding author.
